# Architects of Pituitary Tumour Growth

**DOI:** 10.3389/fendo.2022.924942

**Published:** 2022-06-28

**Authors:** Maria Eugenia Sabatino, Ezequiel Grondona, Ana Lucía De Paul

**Affiliations:** ^1^ Universidad Nacional de Córdoba, Facultad de Ciencias Químicas, Córdoba, Argentina; ^2^ Consejo Nacional de Investigaciones Científicas y Técnicas, Instituto de Ciencia y Tecnología de Alimentos Córdoba (ICYTAC), Córdoba, Argentina; ^3^ Universidad Nacional de Córdoba, Facultad de Ciencias Médicas, Centro de Microscopía Electrónica, Córdoba, Argentina; ^4^ Consejo Nacional de Investigaciones Científicas y Técnicas, Instituto de Investigaciones en Ciencias de la Salud (INICSA), Córdoba, Argentina

**Keywords:** pituitary gland, tumour growth, tumour growth suppression, cellular fates, cellular physiology

## Abstract

The pituitary is a master gland responsible for the modulation of critical endocrine functions. Pituitary neuroendocrine tumours (PitNETs) display a considerable prevalence of 1/1106, frequently observed as benign solid tumours. PitNETs still represent a cause of important morbidity, due to hormonal systemic deregulation, with surgical, radiological or chronic treatment required for illness management. The apparent scarceness, uncommon behaviour and molecular features of PitNETs have resulted in a relatively slow progress in depicting their pathogenesis. An appropriate interpretation of different phenotypes or cellular outcomes during tumour growth is desirable, since histopathological characterization still remains the main option for prognosis elucidation. Improved knowledge obtained in recent decades about pituitary tumorigenesis has revealed that this process involves several cellular routes in addition to proliferation and death, with its modulation depending on many signalling pathways rather than being the result of abnormalities of a unique proliferation pathway, as sometimes presented. PitNETs can display intrinsic heterogeneity and cell subpopulations with diverse biological, genetic and epigenetic particularities, including tumorigenic potential. Hence, to obtain a better understanding of PitNET growth new approaches are required and the systematization of the available data, with the role of cell death programs, autophagy, stem cells, cellular senescence, mitochondrial function, metabolic reprogramming still being emerging fields in pituitary research. We envisage that through the combination of molecular, genetic and epigenetic data, together with the improved morphological, biochemical, physiological and metabolically knowledge on pituitary neoplastic potential accumulated in recent decades, tumour classification schemes will become more accurate regarding tumour origin, behaviour and plausible clinical results.

## Introduction

### Pituitary Tumorigenesis Unintelligibility

The pituitary is a master gland responsible for the modulation of critical endocrine functions. This entails a subtle responsiveness to dynamic cell signalling, resulting in a fine physiological adjustment for homeostasis preservation. Pituitary neuroendocrine tumours (PitNETs) display a considerable prevalence of 1/1106 in the general population (1), which are frequently observed as benign solid tumours.

Traditionally, it has been proposed that anterior PitNET growth starts from a monoclonal origin and propagates slowly, becoming more aggressive while transiting from microtumour to early macrotumour and, subsequently, expressing an invasive profile that eventually transforms into a carcinoma *via* metastasis (2). Angiogenesis and invasive performance are presumed to be determinant for pituitary carcinoma development, and are assessed by VEGF, EGF, COX-2, HIF-1a expression and RSUME up-regulation (2–4).Histologic typification is the most common method used by far for determining particular growth patterns and prognosis. The actual WHO classification provides detailed histological subtyping of a PitNET, based on the tumour cell lineage, cell type, and related characteristics (5). Yet, no reliable aggressive predictor has been defined for most PitNET, although some histologic subtypes can present aggressive behaviour (6, 7). The high-risk PitNETs recognised are: sparsely granulated somatotroph tumours, lactotroph tumours in men, Crooke’s cell tumours, silent corticotroph tumours, and Pit-1 positive plurihormonal tumours (8). The driveline responsible for the rarely invasive and metastatic profile is unclear, even when oncogenic pathways are triggered or tumour suppressor pathways are deactivated.

The apparent scarceness, uncommon behaviour and molecular features of PitNETs has resulted in a relatively slow improvement in depicting their pathogenesis. A better understanding of the several phenotypes or cellular responses during tumorigenesis is needed, as histological typification has a limited forecasting potency, although it is still the main option for prognosis determination.

### New Approaches for Pituitary Tumorigenic Models

Despite their generally benign growth, PitNETs still represent a cause of important morbidity due to hormonal systemic deregulation, which requires surgical, radiological or chronic treatment for illness management (1, 9). Most PitNETs appear as macrotumours (1, 10), which are submitted to surgical removal as the first-line treatment, except for dopamine agonist treated lactotroph tumours (7, 9). However, entire surgical resection can be arduous due to the pituitary location. In addition, considering that PitNETs show relatively lower remission rates (10, 11), novel models with greater forecasting efficacy continue to be necessary.

Currently, PitNET diagnosis and classification are in routine practice still based on hormone immunohistochemical examination. As every cell type within the pituitary gland is able to yield tumours, a varied group of neoplasms can occur, usually related to overly secreted hormones. This subclassification could become more complex since it is now recognised that there are also tumours with varied secretory properties, either due to plurihormonal or multicellular pituitary neoplasia (8, 12, 13). Some of these discrepancies have been resolved by molecular studies, through typification of tumour origin by the transcription factors involved in the differentiation of anterior pituitary cells, with PIT1, TPIT, SF1, GATA3, and ERα providing histological subtyping of PitNETs (9). However, the numerous stages of pituitary tumorigenesis are still poorly understood.

We envisage that through a combination of molecular, genetic and epigenetic data, together with morphological, biochemical, physiological and metabolically knowledge on pituitary neoplastic potential accumulated in recent decades, tumour classification schemes will become more enlightened regarding tumour origin, behaviour and plausible clinical results (14, 15). Thus, with the aim of improving pituitary tumorigenesis aetiology comprehension, new integrative perspectives should help to unravel the underlying mechanisms of PitNET tumour growth.

### Models of Unconstrained Cell Proliferation in Pituitary Neuroendocrine Tumours

Many efforts have been made to identify the mechanisms and agents involved at either the beginning or evolution of PitNETs. Typical oncogene mutations such as Ras or p53 genes, have not been effectively associated with unconstrained pituitary cell proliferation (16–19). As an alternative, other reports have pointed out that PTTG, abundantly expressed in most human PitNETs, with its role in initial pituitary tumorigenesis having been experimentally established and related to invasiveness, recurrence, metastasis (20–26). Subsequent reports have recognized PTTG as being the human homolog of securin, which acts in sister chromatid separation during mitosis (27), thereby accounting for PitNET aneuploidy (28). Nevertheless, no substantial association has been determined between PTTG expression and tumour size, grade, or even prognosis or treatment responses (25, 29). Related to this, unconstrained PitNET growth has also been associated with disrupted cell cycle regulation through the alteration of cyclins D1, D3, and E, or cyclin-dependent kinase inhibitor family-like CDKN1A (p21Cip1), CDKN1B (p27Kip1), CDKN2A (p16INK4a p14Arf), CDKN2B (p15INK4b), CDKN2C (p18INK4c) and pRb expression (7, 30–33).

### Cell Death Contribution to Pituitary Neuroendocrine Tumour Development

Despite there being few reports referring to apoptosis in PitNETs, its contribution in tumour growth and as a prognosis biomarker have been explored (34, 35). Although apoptotic cells are practically absent or difficult to identify in PitNETs (36, 37), a greater apoptotic activity has been reported in aggressive and drug-resistant tumours (34, 38–41), particularly in corticotroph tumours (39, 40). However, since no association with growth rate or recurrence has been noted, there is no support for using the apoptosis index as a prognostic indicator (29, 37). On the other hand, from a molecular point of view, deregulation of apoptosis-related proteins might be a relevant marker of tumorigenesis, with the BCL2/BAX ratio having been proposed (34, 42, 43).

A programmed non-apoptotic cell death was described in PitNET, paraptosis or parapoptosis induced by EGF in a pituitary cell line (44), or by bromocriptine in experimental tumours (45). Furthermore, “dark cells”, referring to a cell death type thus named due to its electron-dense morphological features, was described in dopamine-treated lactotroph turmors and oncocytomas, alongside apoptotic cells (40) and in functional pituitary glands (46). Furthermore, bromocriptine treatment also provokes another cell death type recognised as programmed necrosis or necroptosis, in human lactotroph turmours and in a pituitary cell line (47, 48). To date, however, little information has been combined regarding different types of cell death in the regulation of PitNET formation and progression. Moreover, certain contradictions about the precise role of cell death pathways in pituitary tumorigenesis require further elucidation (7, 29, 37). Thus, apoptosis has not been shown to be a decisive factor in PitNET growth.

### Beyond “to Live or to Die” Cellular Decisions During Tumorigenesis

With the aim of understanding tumorigenesis, many models have principally projected two major cellular fates leading to an extended dichotomised analysis: to proliferate or to die, usually presenting both as excluding outcomes (49–59). Current knowledge has established the intricacy of the signalling networks that guide and preserve tumours, implying coordination of the intra- and extracellular cues that trigger various pathways, either simultaneously or in a spatio-temporal dynamic. Cell survival and proliferation are interrelated with cell death, acting as combined interdependent processes at several points by molecular links responsible for the coordination of cell growth (53, 60, 61). Remarkably, tumour cells harbour the possibility of eliciting intrinsic suppressor programmes, thus permitting tumour progression once this interlinked molecular network between proliferation and growth suppression gets uncoupled (62). Consequently, several mechanisms might be triggered to thwart uncontrolled cell division, such as autophagy, cellular senescence, programmed cell death and necrosis; all of which actually appear as crucial responses to tumoral alterations (61, 63, 64).

Pituitary neoplasm behaviour presents a significantly inconsistent and unpredictable growth performance (7–9, 14, 15, 29), so that events such as mitosis and apoptosis have ended up being unhelpful measures (36, 52), thereby requiring models based on proliferation and cell death to be reconsidered.

Achieving successful tumour development not only entails sustained cell division, but also their survival and thriving, circumstances that may require cellular physiology and metabolic reprogramming to cope with a changing environment and cellular damage (62). All these adjustments might transform a unified group of cells into a small tumour ecosystem, in which different cell phenotypes compete and may eventually collaborate for available space and resources (65). Accordingly, tumour progression represents the coevolution of a heterogeneous group of cells, which instead of acting as an individual uncontrolled cell, needs to coordinate and obtain the cooperation of the neighbourhood (66).

Evidence accumulated about pituitary tumorigenesis reveals it involves more than just the rate of cell multiplication and loss, with it also depending on more than a univocal abnormality factor in a central proliferation pathway (7). PTs can display intrinsic heterogeneity and cell subpopulations with diverse biological, genetic and epigenetic particularities, including tumorigenic potential (15, 55, 67). Hence, PitNET growth understanding requires new approaches and systematisation of the available data.

The role of cell death programs, autophagy, stem cells, cellular senescence, microenvironment, inflammation, mitochondrial function and metabolic reprogramming are still emerging fields in pituitary research. Future morphological and molecular studies also need to establish spatio-temporal dynamics, cellular heterogeneity, cell physiology adaptation and the ability to cope with cellular damage. To date, few studies have been based on integrative analysis, where proteomic data is used for defining which cell-signalling and metabolic pathways could be the most relevant during PitNET pathogenesis (14, 68–72).

### Genetic and Epigenetic Regulation in Pituitary Neuroendocrine Tumours

Whole genomic sequencing has exposed numerous mutations in PitNETs. However, they display comparatively less genetic anomalies than other tumour types or cancers (73). In general terms, genetic anomalies associated with PitNET tumorigenesis progression may not be conclusive (73). Consequently, as only a small number of pituitary neuroendocrine tumours may be correlated with recurrent somatic mutations and unusual hereditary variations (74), some evidence has suggested that epigenetic modifications may participate in pituitary tumorigenesis (73, 75, 76).

Several reports have identified epigenetic modifications in PitNETs, and DNA methylation has been designated a major strategy for epigenetic modification, (77–79) in addition to the aberrant expression of DNMT enzymes (80, 81). However, even if increased amounts of methylation could be connected with more aggressive PitNETs (73), a correlation between gene expression and promoter methylation may not always be detected (74).

Recent studies have explored multiple dysregulated histone acetylation in PitNETs (82–85), which may lead to acetylation of the PTTG promoter (86), suggesting the presence of different arrangements of histone modifications (73). In addition, fluctuations in miRNA expression in several tumours reveal that this kind modification could be involved in essential decisions throughout tumour progression (87, 88). Indeed, altered miRNA expression has been shown to be related with increased or decreased tumour diameter, invasiveness, tumour subtype and therapeutic outcomes (89–97).

### Coping With Cellular Stress: Cellular Physiology and Metabolic Status in Pituitary Neuroendocrine Tumour Cells

#### DNA Damage

Genomic instability is an extended feature of almost all tumour cells (98), Concerning pituitary neoplasia, it has been stated that genomic instability and oxidative DNA damage often occurs and could be associated with an early biomarker of invasive and aggressive behaviour (99–101). Moreover, loss of heterozygosity and an altered number of somatic copies of genes were reported in secreting and aggressive sporadic pituitary neuroendocrine tumours, and may correlate with clinical phenotypes (99, 102–104).

DNA repair systems is a main constituent of DNA damage response (DDR) in normal conditions, with ineffectiveness in this process being linked with the susceptibility to tumour growth through the occurrence of genomic instability (105, 106). Conversely, DDR also serves as a physiological barrier against tumour initiation or progression (107, 108). However, information about the DDR contribution to pituitary tumorigenesis is scarce. Gene mutations involved in DNA mismatch repair have been recognised in Lynch syndrome patients presenting aggressive corticotropin-secreting tumours (109, 110). Also, missense mutations have also been reported in mismatch repair genes in non-secreting tumours (111). DNA damage signs have appeared in association with cellular senescence in somatotroph tumours (112) and also in experimental lactotroph turmors (113). Moreover, PTTG was related to aneuploidy and DNA damage senescent GH-secreting cells, which are potentially responsible for growth constraint (114). Recently, it was shown that cAMP and Fanconi anemia DNA damage repair pathways were affected by alterations in the somatic copy number in somatotroph tumours, which could act as pathogenetic drivers of tumorigenesis (104).

#### Cellular Bioenergetics

The mitochondria’s mandatory role as an energy provider establishes it as a crucial link of cellular metabolism and oxidative stress management, thus supporting processes such as proliferation, apoptosis, autophagy, senescence and immunity response (115). In addition, mitochondrial proteins can regulate numerous signalling pathway networks and cellular behaviours, with this organelle being involved in an extensive range of diseases, including tumorigenesis (116). Molecular network studies have revealed that mitochondrial dysfunction, oxidative stress and mitochondria-mediated ROS-mitogen-activated protein kinase (MAPK) signalling abnormality are significantly associated with the pathogenesis of PitNETs (69, 70, 72, 117–120).

The modification in energy metabolism needed for tumour formation or progression is related to mitochondrial adaptation and seems to play an imperative role in PitNETs by influencing cell proliferation, growth, and angiogenesis. Increases in the mitochondria number (121–124), fusion process and biogenesis have been found during experimental pituitary tumorigenesis (124), with the volume of mitochondria varying between diverse tumour subtypes (118). In addition, an augmented production of lactate dehydrogenase toward aerobic glycolysis (124, 125) and a modification in the fatty acid metabolism have also been observed (97).

Amplified ROS and RNS actions and oxidative stress have been regarded as critical contributors in the pathogenesis of PitNETs (124–130). Furthermore, mitochondrial dysfunction has been described showing morphological and functional changes, such as bigger mitochondria with irregular swelling and fragmented cristaes (124). An activation of the nuclear factor erythroid 2 like 2 (Nrf2) pathways, a main regulator of oxidative stress response along with oxidative damage signal reduction, have been reported during PitNET development, which might provide cellular survival advantages (120, 124). Indeed, elevated mitophagy and mitochondrial dysfunction may favour resistance to chemotherapy in the pituitary GH3 cell line (131). Conversely, activation of mitochondria-mediated apoptosis has been proposed, which might favour novel therapy drugs (132–134). Although this evidence validates the significant roles of mitochondrial functions and adaptability in pituitary tumorigenesis, their molecular mechanisms still need to be clarified.

### Surviving or Thriving: Senescence and Autophagy in Pituitary Neuroendocrine Tumours

#### Cellular Senescence

Cellular senescence (CS) is considered a stress response determined by stable cell cycle arrest in which cells remain viable and metabolically active (135, 136), with many studies having reported the presence of CS in PitNETs. As it is understood to be a spontaneous initial barrier in tumorigenesis (137), CS might constitute a conceivable explanation for the slow and benign growth of PTs (2, 138).

Significant differences in CS marker expression have been detected in human PitNETs (139–141). Moreover, a certain specificity of this cellular phenomenon to the tumour subtype has been suggested, as frequently aggressive ACTH tumours present lower senescence signs (141). Several experimental models have reported the contribution of CS during pituitary tumorigenesis (112–114, 142–144), supporting the idea it may be an impediment against oncogenic stimulation and prevent cellular transformation (145).

The driver forces underlying pituitary senescence are not entirely deciphered, as various cellular pathways and cytokines seem to contribute in triggering and modifying CS acquisition, such as PTTG, which displays oncogene activities and is overexpressed in many PitNETs (146). Yet, its deletion or overexpression promotes pituitary p53/p21-dependent senescence in GH-secreting cells (22, 112, 114, 147). Furthermore, the involvement of tissue-specific pathways has been proposed of intra-nuclear p21Cip1 diverse expression, p16Ink4a and p15Ink4b (112, 114, 139–141).

CS also develops a complex senescence-associated secretory phenotype (SASP) that emulates an inflammatory response. In particular, IL-6 contributes to maintaining pituitary senescence during tumorigenesis by its autocrine action, providing an IL-6-mediated benign tumour senescence model (67, 113, 138, 144, 148, 149). However, detrimental functions of senescent cells have also been uncovered in cancer development, mainly through pro-tumorigenic factor secretion inducing paracrine tumorigenesis (150). Indeed, pituitary IL-6 may provoke contrary effects (inhibitory or stimulatory) in different tumours such as ACTH-, PRL-, GH-secreting and non-functioning tumours (148). Furthermore, paracrine IL-6 triggered by surrounding folliculo-stellate cells is capable of stimulating tumour development (151).

#### Autophagy

Autophagy is an intracellular catabolic pathway based on self-degradation and recycling of the cellular components that collaborate to physiological homeostasis. However, as autophagy is also implicated under pathological conditions, it is considered a ‘double-edged sword’ for being a tumour suppressor as well as a pro-survival factor (152–154). It is triggered in response to a variety of stimuli and also connected with CS, due to both factors protecting cells from external and internal stressors (63). Autophagy could also be permissive for tumour survival in the face of stress (155, 156).

Concerning autophagy research in PitNETs, crinophagy, a specific form of autophagy of secretory granules, has been reported to have a role in intracellular hormone level modulation (157–159). Autophagy participates in lactotroph turmors cell survival and proliferation by the action of a long non-coding RNA CLRN1-AS1 affecting the Wnt/β-catenin signalling pathway (160).

Several reports have related autophagy to PitNET clinical behaviour and drug therapy sensitivity (161, 162). Dopamine agonists, cabergoline and bromocriptine, the first choice treatments for lactotrophs tumours, trigger autophagy in tumoral cells (163–165). Also, somatostatin analogue (octreotide, lanreotide or pasireotide) treatment induces concomitantly apoptosis and autophagy in GH tumours of acromegaly patients (166). The contribution of autophagy to radiotherapy and its manifestation in pituitary carcinomas has yet not been examined. Therefore, it is possible to suggest that the role of autophagy in PitNET cell biology might be context-dependent (161, 162, 167), although its mechanism elucidation requires further investigation.

### A Novel Cellular Population Susceptible to Contributing to Pituitary Neuroendocrine Tumours: Pituitary Stem Cells

Resident pituitary stem cells (PSCs) exist at the marginal zone (MZ) of the intermediate lobe, dorsal anterior lobe (AL) and throughout the AL parenchyma (168–170). This group of cells are involved, at least in part, in tissue remodelling and hormone-producing cell generation during embryonic and postnatal life (171–173). This long-term pool of undifferentiated progenitors shares stemness-related factors which confer on them self-renewal and pluripotency properties responsive to homeostatic balance and injury (174–179). In addition, the *in vivo* expression of multiple markers indicates the existence of PSC population subsets or heterogeneity, which act as a cellular niche driving physiological pituitary plasticity (179–181).

Stem cells and cancer stem cells have been described in PitNETs (CSCs) (182–184) displaying SOX2 and NANOG expression, two pluripotency-associated transcription factors (185), possibly representing a tumour-initiating cell population (173, 186). In addition, the presence of plurihormonal and null cell-type tumours and the low mitotic rate present in the hyperplastic pituitary suggest that PSC are a potential cellular source of PitNETs (187). Pituitary neuroendocrine tumour stem cells (PASCs) expressing GFRa2, Sox9, Nestin, CD133 and CD44, identified in normal and experimental PitNETs, along with variations in PSC/CSC marker expression, were notably detected at tumour initiation (184). Further characterizations of human adult PSCs are now necessary to obtain better understanding of the physiological and pathological roles of these cell subsets. Innovative *in vitro* investigations, such as PSC-derived organoid models (188, 189), should provide a deeper insight into the role of PSC/CSC in pathophysiological contexts, thereby contributing to PitNET growth control.

## Perspectives and Final Considerations

Tissues execute a continuous counterbalance between proliferation, differentiation and death in order to preserve a normal and healthy structure and function. These tasks involve an unceasing choreography, as evidenced by cellular architecture, and are coordinated by paracrine interactions. The loss of homeostatic dynamics can arise by mutations, cellular damage or stress, leading to aberrant proliferation, an essential step for tumour formation.

Many decades of tumorigenesis research have been devoted to identifying the genetic and molecular players, central keys or pathways that are mainly responsible for a particular cell fate decision, frequently within the proliferate/die binary axiom. However, the cellular decision process in tumour biology may result from redundant, interconnected and double-edged sword molecular signalling pathways. Biological networks are characterised by multiple feed-forward, feedback, and cross-talk characteristics that compensate for perturbations affecting individual components and provide them with great robustness. These are intricate dialogs entailing soluble molecules that comprise growth factors, cytokines, hormones and proteases, and also insoluble factors such as extracellular matrix components or cell-cell interactions. Figuring out how single components of such a complex and multifaceted network collaborate to the output of each programme network is a key requirement, because analysing components separately cannot provide a whole picture of the network dynamics.

As intratumoral heterogeneity might exist in many forms, the alteration of multiple, sometimes superposed molecular pathways can be condensed and understood as an array of phenotypes or behaviours, which can then be incorporated into a cellular interaction model. This involves not just the determination of individual genetic or epigenetic subtypes, but also the integration of consequent phenotypic features with the microenvironment to reflect this complex interplay, which may help to dissect definable tumour outcomes. We should emphasise the incorporation of cellular physiology and metabolic status and reprogramming examination as a tumour fitness depiction, thereby providing a measure of the ability of tumour cell phenotypes to survive and grow.

**Figure 1:**
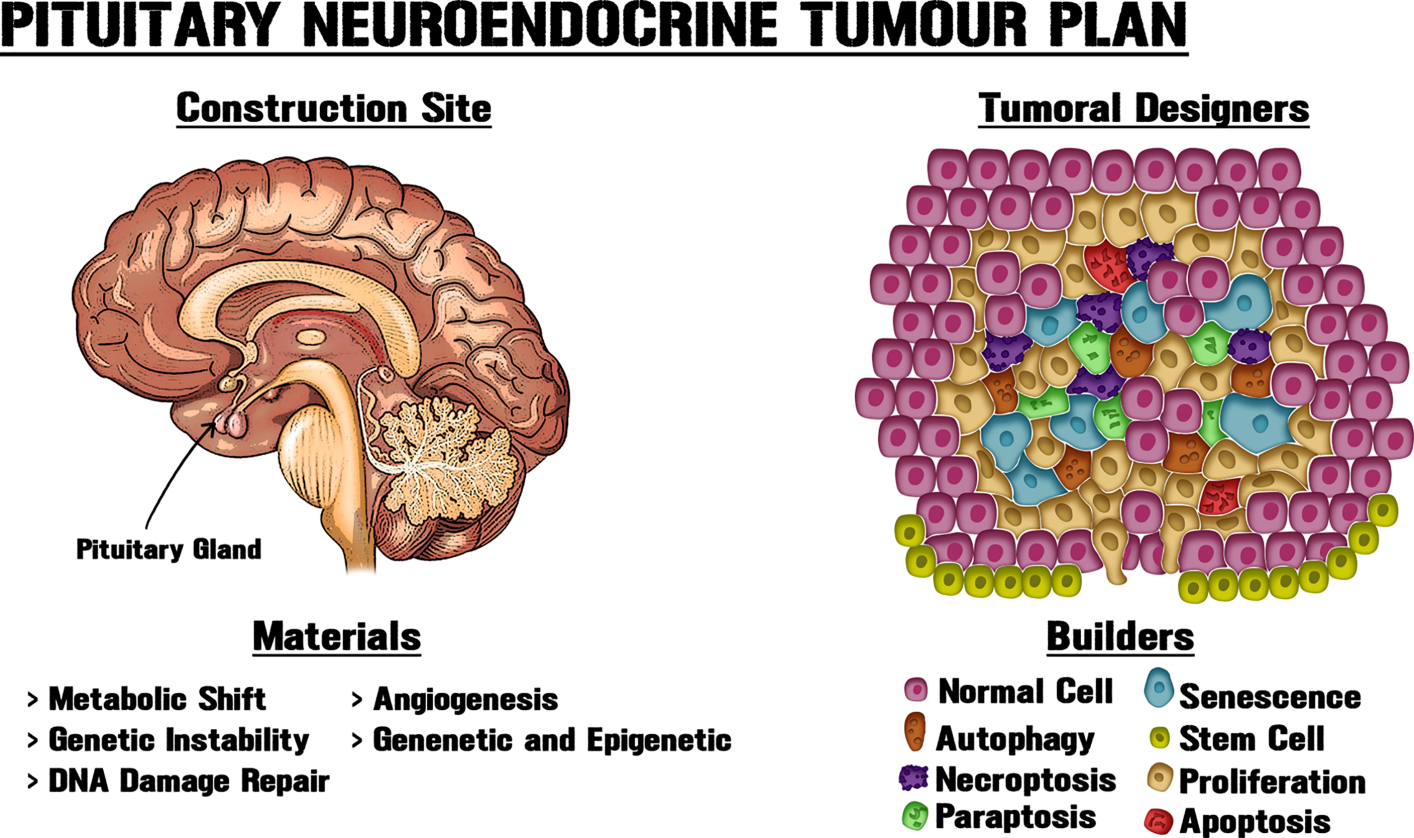
New approaches for pituitary tumorigenic models. PitNETs can display intrinsic heterogeneity and cell subpopulations with diverse biological, genetic and epigenetic particularities, including tumorigenic potential. Tissue architecture and microenvironment could play vital roles in neoplasms. Like an architect who determines the use of materials at a building site, different cellular processes modify the structure and interactions within the gland, thereby shaping tumour growth. Obtaining a better understanding of PitNET growth requires new approaches and systematization of the available data through the combination of molecular, genetic and epigenetic data, together with the utilization of morphological, biochemical, physiological and metabolic knowledge about pituitary neoplastic potential.

We have only lately begun to understand the variety and complexity of machinery by which tumorigenic lesions develop. The deficiency of many long-used models to faithfully represent the complexity of systemic tumour behaviour has generated a greater necessity for combining several viewpoints, to produce a wider comprehension of the critical objective of interventional therapies. By considering an ecological perspective for tumour cells, it is possible to define grouped or collective phenotypes beyond searching for individual mutations. In this cellular ecosystem, the relations among confined contributors will progressively transform, creating a vast net of cellular cross-talking and structural components that can promote growth (**Figure 1**). We suggest that the tissue architecture and microenvironment could play vital roles in neoplasms. New models that envisage a major complexity will be able to generate a tumour fitness interpretation, and allow the gradation of pituitary trophic plasticity to be discerned in order to bring boosted responses to regular stimuli throughout life and to the suboptimal reactions or homeostasis restoration that possibly influence trophic anomalies.

## Author Contributions

MS, EG and ADP contributed to conception and design of the review. MS, EG and ADP organized the articles included. MS wrote the first draft of the manuscript. MS, EG and ADP wrote sections of the manuscript. EG designed the included figure. All authors contributed to manuscript revision, read, and approved the submitted version.

## Funding

This work was funded by Consejo Nacional de Investigaciones Científicas y Técnicas (CONICET PIP 2020-2023 No. 11220200102210), Secretaría de Ciencia y Tecnología, Universidad Nacional de Córdoba (SECyT-UNC 2018-2022 Nº33620180100675CB), and Agencia Nacional de Promoción Científica y Tecnológica - Ministerio de Ciencia y Tecnología (FONCYT-PICT 0950-2020) to ALDP.

## Conflict of Interest

The authors declare that the research was conducted in the absence of any commercial or financial relationships that could be construed as being a potential conflict of interest.

## Publisher’s Note

All claims expressed in this article are solely those of the authors and do not necessarily represent those of their affiliated organizations, or those of the publisher, the editors and the reviewers. Any product that may be evaluated in this article, or claim that may be made by its manufacturer, is not guaranteed or endorsed by the publisher.
